# Effect of resilience on infertile couples’ quality of life: an actor–partner interdependence model approach

**DOI:** 10.1186/s12955-020-01550-6

**Published:** 2020-09-01

**Authors:** Ju-Young Ha, Seon-Hwa Ban

**Affiliations:** College of Nursing, Pusan National University, 49 Busandaehak-ro, Mulgeum-eup, Yangsan-si, Gyeongsangnam-do 50612 South Korea

**Keywords:** Quality of life, Resilience, Infertility, Actor–partner interdependence model

## Abstract

**Background:**

Infertility is an emerging socioeconomic issue affecting an individual’s life and the nation. However, only few studies have examined the influence of resilience on the quality of life (QoL) of spouses as actors and partners. Hence, this study aimed to analyze the influence of resilience, a positive factor that infertile couples may have, on QoL using the actor–partner interdependence model (APIM).

**Methods:**

By the analyses of couples’ data, we analyzed the effect of resilience on the QoL of infertile couples as actors and partners. This cross-sectional study included 150 infertile couples. The Fertility Quality of Life and Resilience Scale was used to measure couples’ QoL and resilience. APIM was applied to analyze QoL.

**Results:**

In terms of actor effects, the resilience of both wives (β = 0.201, *p* <  0.001) and husbands (β = 0.713, *p* <  0.001) had a significant effect on individual QoL. With regard to partner effects, husbands’ resilience (β = 0.351, p <  0.001) had a significant impact on wives’ QoL and the wives’ resilience (β = 0.219, *p* = 0.009) had a significant impact on husbands’ QoL.

**Conclusions:**

The resilience of an infertile actor was found to affect both his/her own QoL and his/her partner’s QoL. In the future, if a program is to be developed to improve couples’ QoL, both spouses should work together to improve their resilience, thereby improving their QoL.

## Background

Infertility is a prevalent condition among reproductive-aged couples worldwide. In Korea, the number of infertility diagnoses increased by approximately 24% from 178,000 in 2007 to 221,000 in 2016 [1]. Infertility-related healthcare costs also increased from KRW 9.575 billion in 2005 to KRW 43.42 billion in 2017 [2]. Thus, infertility is an emerging socioeconomic issue that affects not only individual lives but also the nation.

Because infertility and its subsequent treatment can be a negative psychological burden for infertile couples [3], it is likely to have a significant influence on their quality of life (QoL) [4]. Fertility and problems associated with it affect couples’ QoL, which leads to social and psychological stress, reduced life satisfaction, increased marital problems, and reduced sexual self-confidence as well as decreased sexual and marital satisfaction [5].

In a previous study, resilience was considered as a factor that could positively affect the QoL of infertile couples [6]. Resilience is defined as the extent to which an individual adapts to difficult life events [7]. Moreover, it plays the role of protecting affected couples from stress caused by infertility so that they are able to maintain and enhance their QoL despite the negative impact of infertility [6]. In addition, resilience plays the role of a moderator between infertility stress and QoL [8]. Therefore, to provide help for infertile couples whose QoL is threatened by social and psychological stress due to infertility, studies are needed to examine the relationship between the resilience of infertile couples and their QoL.

In particular, infertility affects both the spouses and not just one [9]. Therefore, it would be useful to analyze these effects using the actor–partner interdependence model (APIM) in which the bidirectional influence of the couple is evaluated and the influence of partners on each other is clarified [10]. Maroufizadeh et al. examined the effects of depression on QoL, whereas Kim et al. confirmed the effects of infertility stress, marital adjustments, and depression on QoL [11, 12]. Only a limited number of studies have examined the influence of resilience on the QoL of spouses as actors and partners [5, 8]. In this study, we used the APIM to understand the actor and partner effects of resilience, a positive psychological factor, on the QoL of infertile couples; resilience is expected to be positively associated with their QoL.

Thus, the present study aimed to (a) evaluate whether there were differences in the level of resilience and QoL between male and female dyads experiencing infertility and (b) use the APIM approach to elucidate and differentiate actor effects and partner effects of resilience on QoL.

## Methods

### Participants and study design

We conducted a cross-sectional study on infertile couples using the convenience sampling method. The inclusion criteria were as follows: 1) couples diagnosed with infertility, 2) those with primary or secondary infertility and without children, 3) those aged > 18 years, and 4) those willing to voluntarily complete a multi-item questionnaire. In contrast, the exclusion criteria were as follows: 1) couples with a history of psychiatric problem and 2) those with other major diseases during the study period. The participants were recruited from August 2018 to October 2018, and the target sample size was 150. The sample size in this study was determined using Song and Kim method, according to which, the appropriate sample size was assumed to be 150–400 [13].

### Data collection

Data were collected from Haeundae Community Health Center in Busan, South Korea, and an online infertility website. The owner of this website is an obstetrics and gynecology specialist, and medical information about infertility treatment is shared in this internet portal community.

Prior to collection, we obtained approval for this study from the director of Haeundae Community Health Center. The researcher approached infertile couples who were visiting the community health center to enroll in the national support program for infertile couples and explained the purpose of the study. The questionnaire was administered to those who agreed to participate in the study. If both spouses were present, they were asked to fill the form separately, and the questionnaires were collected on the spot. If only one spouse was present, he/she was given an envelope containing the study description and questionnaire for the couple to complete at home and bring it on a scheduled date. In total, 97 couples participated in the study and 92 couples provided complete answers to the questionnaires.

A study recruitment notice was posted along with the researcher’s telephone number on the site’s bulletin board of the online infertility website. We explained the purpose of the study over the phone to those who contacted us and wished to participate in the study. The couples were instructed to send a proof of infertility, such as a medical report and doctor’s note, via text message. An envelope including the purpose of the study and a consent form was mailed to those who agreed to participate, and signed consent forms were mailed back to us. After collecting the informed consent forms, an online link for the questionnaire was sent to the couples individually via a text message. The response rates were immediately confirmed on the site in which the survey results were tallied. In total, 60 couples completed the survey and two did not.

The infertile couples were instructed to fill out the questionnaire without discussing the answers with each other. Of the 157 eligible couples, seven declined to participate. Finally, 150 couples completed the survey in the current study (response rate: 95.5%).

The questionnaire took approximately 10–15 min to be completed. After the questionnaire was completed, the subjects were remunerated for their participation in the study.

### Instruments

Consent for the use of tools in this study was obtained from the authors of previous studies.

### Resilience

We used the translated version of the Resilience Scale developed by Wagnild and Young [7, 14]. The scale comprises 25 questions—17 questions on personal competence and 8 on acceptance of self and life. Each item was scored on a 7-point Likert scale ranging from 1 (strongly disagree) to 7 (strongly agree). Higher scores represented a greater resilience. The reliability coefficient Cronbach’s α values were 0.85 for the scale originally developed, 0.88 for that used in Song Yang-sook’s study, and 0.80 for that used in the current study [14].

### FertiQoL questionnaire

FertiQoL is a self-administered questionnaire used to assess QoL among infertile individuals [15]. The FertiQoL, translated by Kim Joo-hee, was utilized [16]. This scale comprises two modules—the Core FertiQoL and Treatment FertiQoL. The optional Treatment module was not used in the current study. The Core FertiQoL module yields four subscales, which are as follows: emotional, mind–body, relational, and social. Each subscale comprises six items, and the respondents answered each item using a 5-point Likert scale. The raw total scores and the subscales scores were scaled, ranging from 0 to 100. A higher score represents a better QoL.

The reliability coefficient Cronbach’s α values were 0.92 (approximately 0.75–0.90) for the scale originally developed, 0.92 (approximately 0.72–0.89) for that used in Kim Joo-hee’s study [16], 0.91 for that used in Maroufizadeh’s study [11], 0.93 for that used in Li’s study [8], and 0.88 (approximately 0.68–0.71) for that used in the current study.

### Demographic characteristics of the couples

Data on the demographic characteristics of the couples, such as age, length of marriage, duration of infertility, national financial support, cause of infertility, and psychiatric treatment, were collected using a questionnaire.

### Statistical analysis

The data collected were analyzed using Statistical Package for the Social Sciences (SPSS) Version 22.0 and Analysis of Moment Structures (AMOS) Version 22.0. Descriptive statistics on the demographic characteristics of the participants and measurement variables of the couples were presented using SPSS descriptive statistics, and the skewness and kurtosis of the measurement variables were examined to test the normality of the data.

In addition, Pearson’s correlation coefficients were used to check the correlation and multicollinearity of each factor and the measurement variables. To identify the actor and partner effects of infertile couples’ resilience on their QoL, we used the AMOS structural equation model. We chose the structural equation model because it has the advantage of statistically comparing and evaluating the magnitudes of the estimates obtained through model verification.

Confirmatory factor analysis was conducted to investigate the validity of the latent variables for the model. The fit of the model was evaluated using absolute goodness-of-fit indices and incremental fit indices. Absolute goodness-of-fit indices included chi-square test (χ^2^), χ^2^/df, root-mean-square error of approximation (RMSEA), standard root-mean-square residual (SRMR), goodness-of-fit index (GFI), and adjusted goodness-of-fit (AGFI). Incremental fit indices included an incremental fit index, comparative fit index (CFI), normed fit index (NFI), incremental fit index (IFI), and Tucker–Lewis index (TLI). Finally, bootstrapping in AMOS was used to verify the statistical significance of the paths in the structural equation model.

## Results

### Demographic characteristics of the infertile couples

Table 1 presents the demographic characteristics of the infertile couples. The average ages were 35.81 years for the husbands and 34.03 years for the wives (t = 13.95, *p* <  0.001). None of the couples received psychiatric treatment. In 64.2% of the couples, the average length of marriage was 4–6 years. Moreover, the average duration of attempted pregnancy was 5 years in 29.8% and 4 years in 25.2% of the couples. About 90.1% received national support for infertility treatment. According to medical reports, the cause of infertility in 90.7% of the couples was idiopathic (unexplained).

**Table 1:** Demographic characteristics of the couples (*n* = 150 couples)

		Couples
		n(%)
Duration of marriage	1–3 years	30 (20.5)
	4–6 years	97 (64.2)
	7–9 years	23 (15.2)
Duration of infertility	2 years	29 (19.2)
	3 years	25 (17.2)
	4 years	38 (25.2)
	5 years	45 (29.8)
	6 years	13 (8.6)
National support	Yes	135 (90.1)
	No	15 (9.9)
Cause of infertility	Unexplained	136 (90.7)
	Woman	8 (5.3)
	Man	6 (4.0)

### Resilience and QoL of male and female dyads

As presented in Table 2, the wives had lower resilience (111.26 vs. 120.36; *p* <  0.001) and QoL (65.63 vs. 72.23; p <  0.001) than the husbands. Moreover, with respect to the subscales of FertiQol, the wives had significantly lower scores than the husbands in all domains of QoL.

**Table 2:** Level of resilience and quality of life among infertile couples (n = 150)

Variables	Wives	Husbands	t value	*p* value
Resilience	111.26 (± 16.61)	120.36 (± 12.31)	5.41	< 0.001
Personal competence	80.59 (± 11.22)	82.87 (± 9.97)	1.94	0.052
Acceptance of self and life	30.67 (± 6.23)	37.50 (± 4.41)	10.98	< 0.001
Quality of life	65.63 (± 9.90)	72.23 (± 11.65)	5.30	< 0.001
Emotional	54.08 (± 13.83)	64.77 (± 14.78)	6.48	< 0.001
Mind–body	67.31 (± 12.30)	70.13 (± 13.05)	1.93	0.054
Relational	69.43 (± 11.47)	77.13 (± 11.71)	5.77	< 0.001
Social	71.70 (±11.41)	76.89 (± 12.80)	3.71	< 0.001

M ± SD = mean ± standard deviation

### Test of measurement model

In order to understand how the measurement variables used in this study account for the latent variables, we tested the measurement model in terms of the resilience and QoL of the husband and wife. The results were as follows: χ^2^ = 68.763 (*p* <  0.001); degree of freedom (df) = 47; χ^2^ /df = 1.463; NFI = 0.930; GFI = 0.936; AGFI = 0.893; CFI = 0.976; IFI = 0.977; TLI = 0.933; RMSEA = 0.056; and SRMR = 0.052. The value of the χ^2^/df index is ≤3. SRMR, a standardized RMR value, is acceptable if the value is ≤0.08 [17]. RMSEA, which considers both model error and simplicity at the same time, is considered appropriate if it is ≤0.08. NFI, the standard GFI, is considered appropriate if it is ≥0.80. GFI and AGFI numbers are appropriate if better than 0.90 [18]. Incremental fit indices, CFI, IFI, and TLI estimates are good if they are ≥0.9, being close to 1 [17].

### Test of the structural model

In order to find the actor and partner effects of couples’ resilience on QoL, the normality of the measured variables was evaluated before a structural equation was modeled. To test the univariate normality of the measured variables, the skewness and kurtosis were calculated. The assumption of normal distribution was satisfied because skewness was ≤3 and kurtosis was ≤10 for both husband and wife. Furthermore, as shown in Table 3, correlations ranged from − 0.012 to 0.803. Only the subscales of similar scales had a high correlation coefficient at 0.803, and it was found between the social domain and the relational domain, which are both the subscales of FertiQoL. To determine multicollinearity, a simple regression analysis of the effects of social and relational domains on QoL was performed. The results showed a tolerance limit of 0.356, variance inflation factor of 2.810, and Durbin–Watson value of 1.982. Thus, the issue of multicollinearity was eliminated [19].

**Table 3:** Correlation between resilience and quality of life among infertile couples (n = 150) *p*-value: < 0.05 *, < 0.01**.

	1	2	3	4	5	6	7	8	9	10	11	12
Wives’ resilience												
Personal competence	1											
Acceptance of self and life	0.79**	1										
Wives’ quality of life												
Emotional	0.26**	0.27**	1									
Mind–body	0.20*	0.23**	0.61**	1								
Relational	0.30**	0.23**	0.44**	0.55**	1							
Social	0.35**	0.29**	0.42**	0.41**	0.68**	1						
Husbands’ resilience												
Personal competence	0.12	0.17*	0.10	0.24**	0.31**	0.34**	1					
Acceptance of self and life	0.17*	0.25**	0.14	0.19*	0.27**	0.25**	0.65**	1				
Husbands’ quality of life												
Emotional	0.21**	0.17*	0.14	0.24**	0.25**	0.35**	0.37**	0.38**	1			
Mind–body	0.23**	0.27**	−0.01	0.06	0.03	0.17*	0.20*	0.17*	0.56**	1		
Relational	0.26**	0.22**	0.15	0.24**	0.26**	0.31**	0.34**	0.30**	0.77**	0.57**	1	
Social	0.25**	0.24**	0.24**	0.31**	0.36**	0.43**	0.42**	0.35**	0.74**	0.57**	0.80**	1

The model has the following characteristics: χ^2^ = 68.763 (*p* <  0.001), df = 47, χ^2^/df = 1.463, NFI = 0.930, GFI = 0.936, AGFI = 0.893, CFI = 0.976, IFI = 0.977, TLI = 0.966, RMSEA = 0.056, and SRMR = 0.052. Based on these characteristics, the model was found to have a good fit, as shown in Table 4.

**Table 4:** Effect coefficients for hypothetical model (n = 150 couples)

		Wives			Husbands		
		Β (SE)	t value	p value	Β (SE)	t value	p value
Total Quality of life	Actor’s resilience	0.201 (0.060)	3.370	< 0.001	0.713 (0.152)	4.685	< 0.001
	Partner’s resilience	0.219 (0.084)	2.607	0.009	0.351 (0.102)	3.428	< 0.001
Emotional	Actor’s resilience	0.330 (0.110)	3.015	0.003	0.959 (0.245)	3.913	< 0.001
	Partner’s resilience	0.096 (0.125)	0.769	0.442	0.167 (0.156)	1.070	0.285
Mind–body	Actor’s resilience	0.266 (0.117)	2.266	0.023	0.374 (0.221)	1.692	0.091
	Partner’s resilience	0.279 (0.128)	2.178	0.029	0.443 (0.217)	2.040	0.041
Relational	Actor’s resilience	0.248 (0.087)	2.853	0.004	0.448 (0.149)	3.012	0.003
	Partner’s resilience	0.198 (0.079)	2.501	0.012	0.483 (0.153)	3.151	0.002
Social	Actor’s resilience	0.182 (0.062)	2.940	0.003	0.610 (0.165)	3.707	< 0.001
	Partner’s resilience	0.174 (0.077)	2.251	0.024	0.303 (0.106)	2.861	0.004

### Impact of resilience on QoL at the dyadic level

**Fig. 1:**
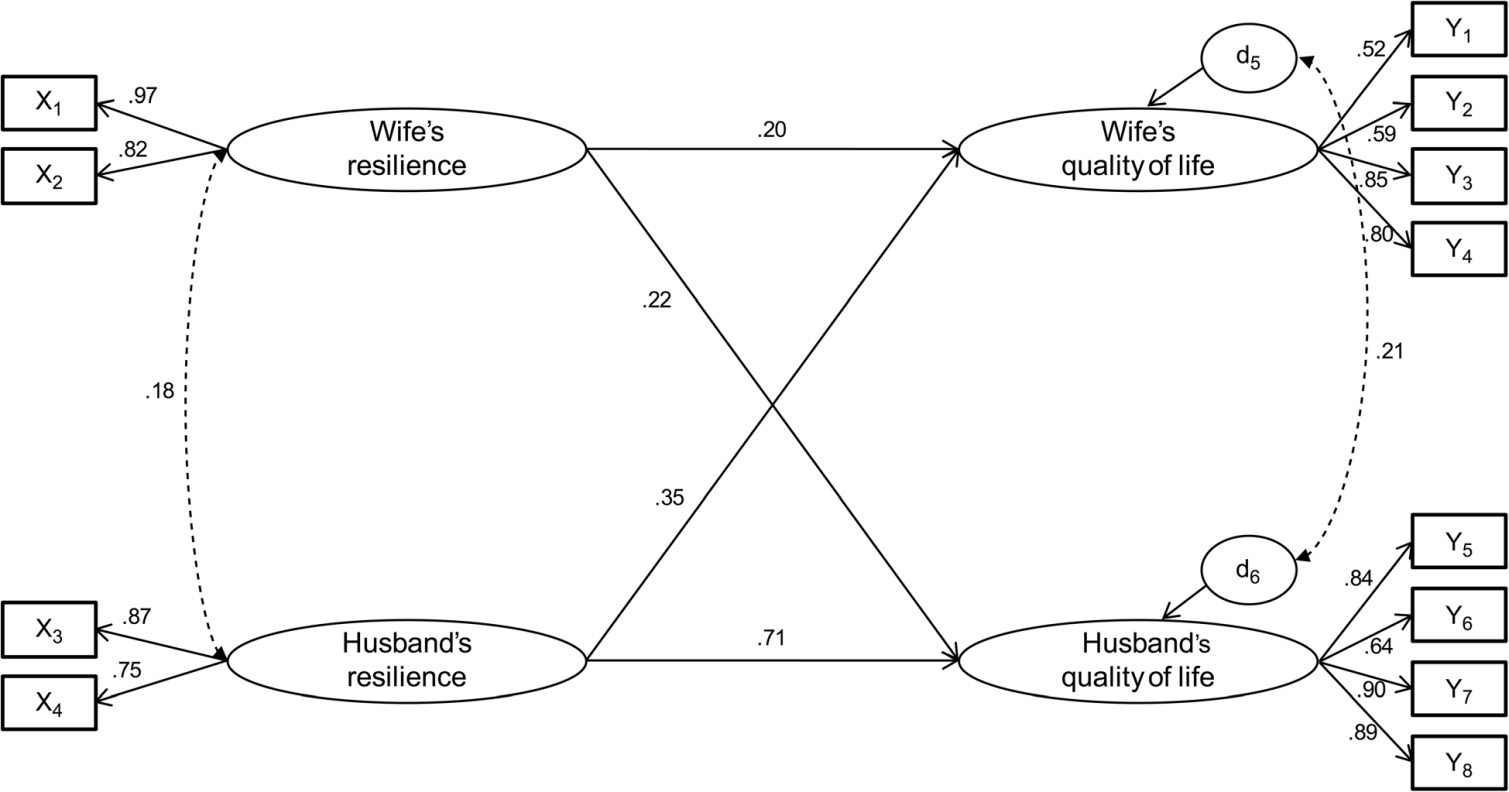
Assessment of the hypothetical model

The results of the actor and partner effects of couples’ resilience on QoL are shown in Table 4 and Fig. 1. The resilience of both wives (β = 0.201, *p* <  0.001) and husbands (β = 0.713, p <  0.001) had a significant effect on individual QoL. Husbands’ resilience (β = 0.351, *p* < 0.001) had a significant effect on wives’ QoL, and wives’ resilience (β = 0.219, *p* = 0.009) had a significant effect on husbands’ QoL.

With regard to actor’s resilience effect on emotional domain, the resilience of both wives (β = 0.330, *p* = 0.003) and husbands (β = 0.959, p < 0.001) had a significant effect on their emotional domain. As for partner’s resilience effect, husbands’ resilience (β = 0.167, *p* = 0.285) had no significant effect on wives’ QoL and wives’ resilience (β = 0.096, *p* = 0.442) had no significant effect on their husbands’ QoL.

With regard to actor’s resilience affecting the mind–body domain, wives’ resilience (β = 0.266, *p* = 0.023) had a significant effect on their mind–body QoL domain, whereas husbands’ resilience (β = 0.374, *p* = 0.091) had no significant effect on their mind–body domain. In terms of partner’s resilience, husbands’ resilience (β = 0.443, *p* = 0.041) had a significant effect on the wives’ mind–body domain and wives’ resilience (β = 0.279, *p* = 0.029) had a significant effect on the husbands’ mind–body domain.

With regard to actor effect on relational domain, both wives’ (β = 0.248, *p* = 0.004) and husbands’ (β = 0.448, *p* = 0.003) resilience had a significant influence on their relational domain. With regard to partner effect on relational life, husbands’ resilience (β = 0.483, *p* = 0.002) had a significant effect on wives’ relational domain, and wives’ resilience (β = 0.198, *p* = 0.012) had a significant effect on husbands’ relational domain.

With regard to the effect of actor’s resilience on social domain, both wives’ (β = 0.182, p = 0.003) and husbands’ (β = 0.610, *p* < 0.001) resilience had a significant effect on their social domain. Regarding partner’s resilience, husbands’ resilience (β = 0.303, *p* = 0.004) had a significant effect on wives’ social domain, and wives’ resilience (β = 0.174, *p* = 0.024) had a significant effect on husbands’ social domain.

In this study, to examine whether the actor effect and the partner effect differed between men and women, these two coefficients were considered equal and were compared using the chi-square test for the constrained and unconstrained (saturated) models (Table 5). As a result of a χ2 difference test between the model constraining the actor effect and partner effect to be equal, which has an impact on the QoL, and the unconstrained model, there was no statistically significant difference in wives (χ2 = 1.823, *p* = 0.177) whereas there was statistically significant difference in husbands (χ2 = 7.654, *p* < 0.01). This implies that for the husbands, the resilience actor effect exerted a bigger impact on the QoL than their wife’s resilience partner effect; and for wives, their husband’s resilience partner effect and their own resilience actor effect exerted an equal level of impact on the QoL. A significant difference was noted between the constrained model in which both the husband and wife’s actor effects were constrained equally and the unconstrained model (χ2 = 10.435, *p* < 0.001). This finding indicated that the husband’s resilience actor effect had a more significant impact on the QoL than the wife’s resilience actor effect. However, there was no significant difference between the constrained model in which the husband and wife’s partner effects were constrained equally and the unconstrained model (χ^2^ = 1.035. *p* = 0.309). This result revealed that the husband’s resilience partner effect and the wife’s resilience partner effect had a similar impact on QoL.

**Table 5:** χ2 Differences in test between the basic model and equivalent constraint model

Model	χ2	df	TLI	CFI	RMSEA	△χ2	p value
basic	68.763	47	0.966	0.976	0.056		
equivalence constraint 1 (a = b1)	76.417	48	0.957	0.969	0.063	7.654	0.010
equivalence constraint 2 (b = a1)	70.586	48	0.966	0.975	0.056	1.823	0.177
equivalence constraint 3 (a = b)	79.198	48	0.953	0.966	0.066	10.435	0.001
equivalence constraint 4 (a1 = b1)	69.798	48	0.967	0.976	0.055	1.035	0.309

CFI = Comparative Fit Index; df = Degree of freedom; RMSEA = Root Mean Squared Error of Approximation; TLI = Turker–Lewis Index; a = Husbands’ actor effects, b = Wive’s actor effects, a1 = Husbands’ partner effects, b1 = Wive’s partner effects

## Discussion

While most previous studies on infertile couples have focused on the effect of negative characteristics on QoL, the current study focused on the effect of resilience, a positive characteristic, on the QoL of infertile couples. In addition, actor and partner effects were identified. Previous studies have evaluated self-identification and relationship satisfaction [20] as well as coping strategies and distress [21] among infertile couples using the APIM approach. However, this is the first study that assessed the relationship between resilience and QoL among infertile couples using the APIM approach.

The QoL of infertile couples measured in this study was standardized for comparison with previous studies. Husbands’ QoL (72.23) in this study was higher than that of wives (65.63), which is similar to the results of 67.36 and 72.89, respectively, for Iranian infertile couples reported in Maroufizadeh et al.’s study and 71.8 and 76.5, respectively, for Turkish infertile couples reported in Goker et al.’s study [11, 22]. This is because wives respond differently; they tend to be more sensitive to and more affected by infertility than husbands [23]. As previous studies have shown, long-term infertility treatment negatively affects women’s emotional domain, subsequently reducing their QoL.

In particular, while the wives’ QoL in this study was lower than that reported in previous studies, it is similar to the 64.54 reported by Li et al. [8]. Infertile women are regarded as defective in Middle Eastern countries, such as in Iran and Turkey; therefore, they are subjected to feelings of failure and inadequacy when they are unable to conceive a child [24]. In Korea and China, where there are strong traditions that emphasize the succession of immediate family, the burden of being unable to carry on the family line is considered a serious problem.

Because resilience refers to the ability to overcome adversity, the resilience that may exist in a couple experiencing infertility is closely associated to their marital relationship [25]. Thus, it is crucial to investigate how resilience affects couples as actors and as partners. Because resilience is an important predictor and a key factor for couples’ marital satisfaction levels [26], it is necessary to focus on couples rather than just individuals. Husbands’ resilience scores were higher than those of wives, similar to the results of Herrmann’s study [6]. Resilience is closely related to positive emotions [27] and a wife facing infertility is more sensitive to negative emotions than her husband [23]. Therefore, husbands who were less exposed to negative emotions had higher scores in resilience than wives.

With regard to effects of resilience on QoL, both wives and husbands showed significant actor and partner effects. These results are similar to those that have been reported in previous studies, showing that resilience was an important factor affecting QoL [6, 28]. Therefore, strengthening resilience is essential for improving the QoL of infertile couples. In addition, resilience affected the QoL of actors and their partners. Therefore, to provide interventions for improving the QoL of infertile couples, it is necessary to strengthen resilience and for spouses to share positive emotions, so that they are able to deal with the various problems involving infertility. Stress caused by infertility can be directly reduced to improve the QoL of infertile couples. However, resilience plays a key role in reducing the impact of stress due to infertility and in maintaining a positive relationship and collective awareness among the couples [29]. Moreover, resilience may be a significant factor as it acts as a mediator between stress caused by infertility and QoL [12].

We could not determine the actor and partner effects on the emotional domain of QoL. The items in the emotional domain include anger, grief, loss, sadness, depression, fluctuating hope, despair, jealousy, resentment, and inability to cope [15], which can be defined as elements that belong to the personal realm representing the private feelings of an individual. In addition, in previous studies, in which resilience was classified as an individual domain [28], husbands’ depression did not significantly affect wives’ QoL [11]. These results are consistent with those reported for the emotional domain in the present study. Further research is needed to clarify the interactions between individual domains and partner effect.

In summary, this study clearly identified the actor and partner effects of infertile couples beyond the correlation between resilience and QoL. Because the actor and the partner influence each other in terms of resilience and QoL, it is necessary to recognize husbands and wives as interdependent beings rather than as independent beings. Furthermore, in intervention studies aimed at improving QoL, a couple should participate as an integrated unit, so that they can seek ways to increase their QoL through their resilience.

Some questionnaires were answered on the spot at the community health center. Meanwhile, some were filled out at the couples’ homes and online. Because the survey was conducted using different methods, it is interesting to evaluate whether this affected the results of the study. There was no statistically significant difference in the mean values between the three groups (wives’ resilience: *p* = 0.960, wives’ QoL: *p* = 0.923, husbands’ resilience: *p* = 0.600, and husbands’ QoL: *p* = 0.359). Nevertheless, future research on the possible impact of the participants’ method of response should be conducted.

This study had several limitations. First, cross-sectional data were used; therefore, a definitive conclusion about causal relationship was not possible. Second, this study only included Korean couples, and this may be a limitation because its results may not be suitably generalized for infertile couples from other cultures and populations. Third, infertility of participants was confirmed via medical reports; however, the cause of infertility specified in the reports was mostly noted to be “unexplained”. Further, participants were either couples who had visited a health center in Busan, South Korea or were members of an online infertility website, indicating that the sample in this study is not reflective of the general population.

## Conclusions

This study employed the APIM approach to identify infertile couples’ resilience on QoL from actor and partner perspectives. The results showed that the resilience of infertile couples affected their QoL both as actors and partners. This study found that spouses mutually influence one another in terms of resilience and QoL; therefore, it is necessary to develop intervention programs involving the participation of couples, and not just individuals, so that they learn to have a positive impact on each other. In particular, it may be necessary to include resilience improvement in both the spouses as an important component in QoL improvement intervention programs.

The results of the current study suggest the following for future research. First, it is necessary to develop intervention programs in which infertile couples participate to improve their resilience and QoL. Second, it is necessary to expand the pool of subjects to include those from other cultures and populations to further investigate the interaction between resilience and QoL using APIM. Third, the current study can be replicated by selecting subjects from wider domains and increasing the sample size to explore the interaction between the individual domain variables and the emotional domain, which is a sub-domain of QoL.

## Data Availability

All data generated or analyzed during this study are included in the published article. For further clarifications, the authors can be contacted at elli2378@hanmail.net.

## Abbreviations

QoL: quality of life
APIM: actor–partner interdependence model
(χ^2^), χ^2^/df: chi-square
RMSEA: root-mean-square error of approximation
SRMR: standardized root-mean-square residual
GFI: goodness-of-fit index
AGFI: adjusted goodness-of-fit index
CFI: comparative fit index
NFI: normed fit index
IFI: incremental fit index
TLI: Tucker–Lewis index
AMOS: analysis of moment structures
SPSS: Statistical Package for the Social Sciences
